# Correction to: ‘Sprinting with prosthetic versus biological legs: insight from experimental data’ (2023) by Beck *et al.*

**DOI:** 10.1098/rsos.230483

**Published:** 2023-05-03

**Authors:** Owen N. Beck, Paolo Taboga, Alena M. Grabowski

*R. Sco. Open Sci.* **9**, 211799. (Published online 5 January 2022). (doi:10.1098/rsos.211799)

The authors of ‘Sprinting with prosthetic versus biological legs: insight from experimental data’ (https://doi.org/10.1098/rsos.211799) published in Royal Society Open Science 5 January 2022 would like to issue a correction to the text provided in the statement of research ethics included with that publication. In the published version of the paper, the authors included the following as a statement of research ethics:

‘The University of Colorado Boulder Biomedical Institutional Review Board (no. IRB00000774) approved the protocol. The participating athlete provided informed consent in accordance with the approved protocol prior to testing (Protocol: 18-0456)’.

Following inquiries by the journal's editorial office to the authors and the Institutional Review Board (IRB) named above, prompted by reader comment, the authors acknowledge that the statement provided is incorrect.

Although informed consent from the study participant was received by the authors, the study did not meet the threshold required by the IRB to be considered ‘research’, and was instead considered a case study. Case studies are not issued an ethical approval (per the guidance at https://www.hhs.gov/ohrp/regulations-and-policy/regulations/45-cfr-46/revised-common-rule-regulatory-text/index.html#46.102 and in the attached PDF), it was therefore incorrect to state that the protocol was approved by the IRB.

The statement on research ethics should more properly have read:

‘The University of Colorado Institutional Review Board (no. IRB00000774) assessed the submitted protocol (Protocol: 18-0456) and determined that it did not meet the definition for research with regard to human subjects. Consequently, no further IRB review nor approval took place or was required prior to the study being conducted. The participating athlete provided informed consent in accordance with best practice’.

The authors are grateful for this opportunity to correct the published statement of research ethics.

